# Changes in the Pulmonary Artery Wave Reflection in Dogs with Experimentally-Induced Acute Pulmonary Embolism and the Effect of Vasodilator

**DOI:** 10.3390/ani11071977

**Published:** 2021-07-01

**Authors:** Tomohiko Yoshida, Ahmed S. Mandour, Katsuhiro Matsuura, Kazumi Shimada, Hussein M. El-Husseiny, Lina Hamabe, Zeki Yilmaz, Akiko Uemura, Ryou Tanaka

**Affiliations:** 1 Department of Veterinary Surgery, Tokyo University of Agriculture and Technology, Tokyo 183-0054, Japan; ruiyue1221@gmail.com (K.S.); hussien.alhussieny@fvtm.bu.edu.eg (H.M.E.-H.); linahamabe@googlemail.com (L.H.); 2 Department of Animal Medicine (Internal Medicine), Faculty of Veterinary Medicine, Suez Canal University, Ismailia 41522, Egypt; 3 VCA Japan Shiraishi Animal Hospital, Saitama 350-1304, Japan; pgwagmw@yahoo.co.jp; 4 Department of Surgery, Anesthesiology and Radiology, Faculty of Veterinary Medicine, Benha University, Moshtohor, Toukh, Elqaliobiya 13736, Egypt; 5 Department of Internal Medicine, Faculty of Veterinary Medicine, Uludag University, Bursa 16120, Turkey; zyilmaz@uludag.edu.tr; 6 Department of Veterinary Surgery, Division of Veterinary Research, Obihiro University of Agriculture and Veterinary Medicine, Hokkaido 080-8555, Japan; anco@vet.ne.jp

**Keywords:** dogs, pulmonary hypertension, pulmonary embolism, right ventricular function, wave reflection, wave intensity analysis

## Abstract

**Simple Summary:**

Pulmonary hypertension (PH) remains a fatal disease, despite the advances in disease-specific therapies. This may be because the assessment of pulmonary hemodynamics in PH has not been established. Recently, several studies have reported that the pulmonary arterial wave reflection (PAWR) might influence the right ventricular afterload and could provide additional information regarding the severity and progression of PH. However, the pathophysiology of PAWR has some unclear points particularly in the case of acute pulmonary embolism (APE). The objective of this study was to investigate, for the first time, the characteristics of PAWR in a dog model of APE using dual-tipped sensor wire. From the result of the present study, after dogs developed PH by injections of dextran microsphere, PAWR was increased significantly along with the pulmonary vascular resistance (PVR) and reduced after vasodilator administration. In addition, PAWR was significantly correlated with PVR and right ventricular fractional area of change (FAC). These results indicating that PAWR may be useful as a new evaluation method in PH and may detect changes related to right ventricular afterload earlier than pulmonary artery pressure (PAP).

**Abstract:**

Pulmonary hypertension (PH) is a complex syndrome that has been frequently diagnosed in dogs and humans and can be detected by Doppler echocardiography and invasive catheterization. Recently, PAWR attracts much attention as a noninvasive approach for the early detection of PH. The present study aims to investigate the PAWR changes in acute pulmonary embolism (APE) and highlight the response of PAWR variables to vasodilator therapy in dogs. For this purpose, anesthesia and catheterization were performed in 6 Beagle dogs. After that, APE was experimentally conducted by Dextran microsphere administration, followed by vasodilator (Nitroprusside; 1μg/kg/min/IV) administration. The hemodynamics, echocardiography, PVR and PAWR variables were evaluated at the baseline, after APE and after administration of nitroprusside. The result showed a significant increase in PVR, PAP, tricuspid regurgitation (TR) as well as PAWR variables following APE induction compared with the baseline (*p* < 0.05). Vasodilation caused by administration of nitroprusside reduced the mean atrial pressure, PVR and PAWR parameters. There were a significant correlation and linear regression between PAWR indices and PVR as well as right ventricular function parameters. In conclusion, PAWR is not only correlated with PVR but also the right ventricular function parameter, which indicates that PAWR may be useful as a new evaluation method in PH, considering that PAWR can assess both right ventricular afterload and right ventricular function.

## 1. Introduction 

Pulmonary hypertension (PH) is a refractory disease that causes right heart failure due to an increase in the pulmonary artery pressure (PAP) and right ventricular afterload [1,2]. In dogs, PH has been recognized as a clinical syndrome for many years, but the routine accurate clinical diagnosis has been enhanced with the widespread usage of Doppler echocardiography in the clinical setting. PH is a multifactorial problem that has been categorized into six groups in dogs based on the disease origin, including pulmonary arterial hypertension, left side heart disease, respiratory disease, pulmonary thromboembolism, parasitic disease and multifactorial or with unclear mechanisms [3].

The right ventricle is vulnerable to afterload especially because of changes in the pulmonary circulation. It is considered that the prognosis of PH often be affected when the right ventricular function is reduced by an increase in the afterload [4,5]. Pulmonary vascular resistance (PVR) and PAP are clinically used as indicators for the severity of PH [6]. The severity of PH is affected not only by PVR and PAP but also by vascular impedance, pulmonary arterial wave reflection (PAWR) and right ventricular function. PVR is only related to the mean component of right ventricular afterload, but nothing about the pulsative changes. Vascular impedance reflects chronic changes with stiffness in the vasculature better [7]. Therefore, PVR or PAP alone cannot truly indicate the severity of PH [8,9,10,11,12].

Recently, several studies have reported that PAWR might influence right ventricular afterload and provide additional information regarding the severity and progression of PH [13,14,15,16,17]. The pulmonary artery (PA) blood flow consists of two waves; forward and reflection. The forward wave is generated toward the peripheral pulmonary artery when the heart contracts [18]. On the other hand, PAWR is a wave that is generated when pulmonary artery blood flow is reflected from the peripheral pulmonary vascular wall. A previous study suggested that PAWR obtained from wave intensity analysis (WIA) was related not only to PVR but also to vascular impedance and right ventricular function [13]. In addition, Castelain et al. also reported that the PAWR may be able to differentiate between chronic pulmonary thromboembolism and PH caused by pulmonary artery hypertension [19]. Therefore, PAWR could provide new information as a severity assessment index of PH. However, the pathophysiology of the PA wave reflection has some unclear points. For instance, the behavior of PAWR in the pulmonary artery embolism did not establish.

The objective of this study was to investigate the characteristics of PAWR in a dog model of APE, highlight the effect of vasodilator administration on the PAWR, and find out the relationship between PAWR and PVR and right ventricular function.

## 2. Materials and Methods

### 2.1. Animal Preparation and Catheterization

Six healthy females Beagle dogs (Kitayama Labes Co., Nagano, Japan) were included in this study (aged 2–3 years; weight 8–12 kg). All dogs were sedated with butorphanol (Vetorfar; Meiji seika pharma, Tokyo, Japan, 0.2 mg/kg, intravenously), midazolam hydrochloride (Dormicum; Astellas Pharma Inc, Tokyo, Japan, 0.2 mg/kg, intravenously), Atropine sulfate (Atropine sulfate; Tanabe Seiyaku Co., Ltd., Saitama, Japan, 25 μg/kg, intravenously) and meloxicam (Metacam 0.5%; Boehringer Ingelheim Vetmedica, Tokyo, Japan, 0.2 mg/kg, subcutaneously). Anesthesia was induced with propofol (Propofol Mylan; Mylan Seiyaku, Tokyo, Japan, 4 mg/kg, intravenously) after tracheal intubation and anesthesia was maintained by isoflurane inhalation (Isoflurane for Animal Use; Intervet, Osaka, Japan, end-tidal concentration of 1.5 ± 0.1%). Heparin sodium (Heparin sodium; Eapharma, Tokyo, Japan, 100 IU/kg, IV) was administered for the prevention of thrombosis. After the dogs were positioned in right lateral recumbency, pressure-controlled ventilation was used to maintain the end-tidal partial pressure of CO_2_ (EtCO_2_) and hemoglobin saturation level of oxygen (SPO_2_) between 35 to 45 mmHg and 95–100%, respectively. Body temperature was kept at 37 °C by using a heat lamp. Following anesthetic induction, a 4 Fr catheter (Atom nutrition catheter; Atom Medical, Tokyo, Japan) was placed in the right femoral artery to measure the mean arterial pressure (MAP). An 18 G peripheral vascular catheter was inserted into the left carotid artery and a 7 Fr vascular introducer sheath (AVANTI^®^+ Sheath Introducer; Cardinal Health Inc, Dublin, OH, USA) was placed inside. After placing the sheath, a 0.035″ × 150 cm guidewire was inserted from the sheath and advanced into the left atrium. After that, 4.2 Fr Multipurpose Angiographic catheter (Goodtec angiographic catheter; GOODMAN CO., LTD, Aichi, Japan) was advanced over the guidewire to the left atrium and placed to measure left atrium pressure (LAP). The used procedures of catheterization did not affect the aortic and mitral blood flow as qualitatively evaluated by echocardiography.

Further, the jugular vein was incised and a 0.035″ × 150 cm guidewire was inserted into the pulmonary artery. A 4.2 Fr MPA catheter was inserted over the guidewire and placed in the pulmonary artery. The combined dual-tipped pressure and Doppler flow sensor wire (Combowire; Royal Philips, Amsterdam, Netherlands) were advanced approximately 1 cm beyond the pulmonary valve. Once stable signals were observed, pulmonary artery pressure (P) and velocity (U) data were acquired simultaneously at a sampling rate of 200 Hz for 30 to 60 s. An additional 4.2 Fr MPA catheter was also inserted from the same incision site of the jugular vein and placed in the right atrium to measure right atrium pressure (RAP). Catheterization-derived measurements

The pressure was measured by the physiological pressure transducer and amplifier system (Life Scope BSM-5192; Nihon Kohden Co., Tokyo, Japan). Cardiac output (CO) was calculated by Fick method using blood sampled from the MPA catheters placed in the left atrium and the right atrium. PVR was calculated as the transpulmonary gradient divided by CO. The transpulmonary gradient was defined as the mean pulmonary artery pressure (mPAP) minus mean left atrial pressure (mLAP). The pulse pressure of the pulmonary artery was defined as the systolic pulmonary artery pressure (SPAP) minus diastolic pulmonary artery pressure (DPAP).

### 2.2. The Study Protocol

**Figure 1:**
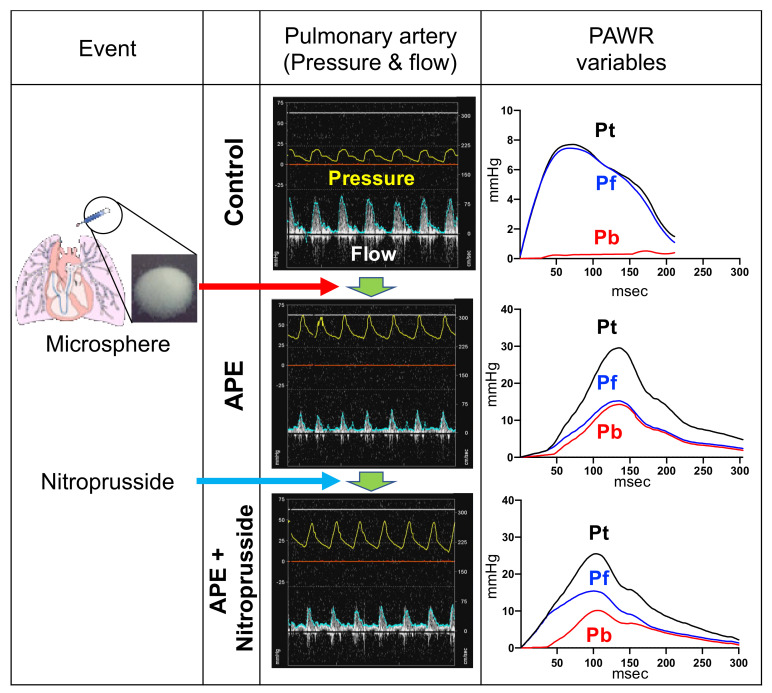
Schematic representation of the used procedures to evaluate pulmonary artery wave reflection (PAWR) wave variables (Pt, total pressure; Pf, forward-traveling pressure; Pb, backward-traveling pressure) after induction of acute pulmonary embolism (APE) in the dog model. The PAWR variables were evaluated at the baseline after anesthesia induction (control), after injection of Dextran microspheres to induce APE and later after administration of nitroprusside to induce vasodilation.

After proper placing and testing of all catheters, the hemodynamic variables and echocardiography were carried out. In addition, the wave reflection was assessed under the following three conditions as showed in Figure 1. (1) Control (Baseline): After the induction of anesthesia, we measured all variables at the baseline. (2) Acute pulmonary embolism (APE). (3) APE and vasodilator administration (APE + Nitroprusside). All measurements have been performed separately under each condition after ensuring the stability of hemodynamics.

### 2.3. Induction of Acute Pulmonary Embolism

Each dog was given repeated injections of dextran microsphere cross-linked with epichlorohydrin (300 μm, Sephadex G-50, GE healthcare, Bio-Science Corp., Uppsala, Sweden) into the inferior vena cava over 5–10 min. The volume of injected microspheres in each dog was adjusted to create a mean PAP above 30 mmHg [20].

### 2.4. Adminstration of Vasodilator

We administered nitroprusside (Nitopro continuous intravenous solution; Maruishi Pharmaceutical Co Ltd., Osaka, Japan) as a vasodilator to dogs that developed PH due to acute APE. Each dog received 1 μg/kg/min/IV [21].

### 2.5. Measurement of the Pulmonary Arterial Wave Reflection

**Figure 2:**
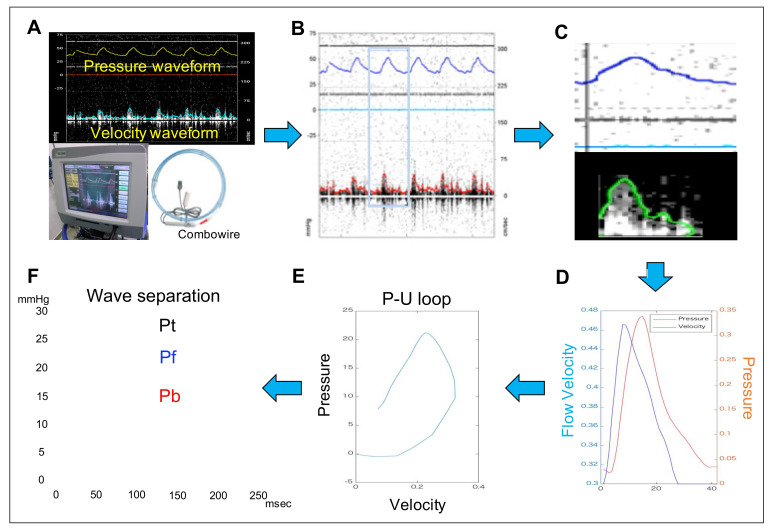
Illustration of the used steps for measurement of pulmonary artery wave reflection parameters. (**A**) After catheterization and establishment of hemodynamic, measuring the pulmonary artery flow and pressure was done simultaneously using combowire. (**B**) Detection of the cardiac cycle. (**C**) Contour detection of pressure waveform and velocity waveform. (**D**) Plotting of pressure and flow velocity. (**E**) Determination of the P-V loop. (**F**) wave separation.

The performed procedures to calculate the PAWR parameters have been illustrated in Figure 2. We can gain PAWR using the concept of WIA [18,22,23]. P-U loop is gained by plotting instantaneous measurements of pulmonary artery pressure and flow simultaneously gained using a dual-tipped sensor wire catheter (Combowire; Royal Philips, Amsterdam, Netherlands). The wave speed (WS) is expressed as the slope of the P-U loop and can take advantage of the water hammer equation relating P and U on the condition that there is no wave reflection in early systole [24]:

c = (dP/dU)/ρ(1)

where dP and dU are the changes in P and U, ρ is the density of blood (1050 kg/m^3^) and c is WS. Pulse pressure can be separated into those attributed to forward-traveling (Pf) [1] and backward-traveling (Pb) [4] waves using Equations (2) and (3).

dPf = (dP + ρc dU)/2(2)

dPb = (dP − ρc dU)/2(3)

where dPf is the temporal change in Pf and dPb is the temporal change in Pb. Pf [5] and Pb [6] can then be determined by summing the differences.

Pf = ΣdPf(4)

Pb = ΣdPb(5)

We can gain 4 wave reflection variables (PAWR variables) from this analysis: peak Pb, peak Pf, WS and reflection coefficient (RC) calculated as the ratio of peak Pb to peak Pf. Pulmonary artery flow and pressure images gained from a dual-tipped sensor catheter were processed using an in-house program code written in MATLAB (Figure 2). The reference P and U waveforms measured directly with the catheter were smoothed using a Savitzky–Golay filter and then ensemble-averaged over three cardiac cycles. These waveforms were then interpolated using a cubic spline and resampled to 1 msec temporal resolution (1000 Hz) [13].

### 2.6. Right Heart Functional Evaluation by Echocardiography

Echocardiography was performed using a ProSound F75 premier CV (Hitachi Aloka Medical Co., Tokyo, Japan) with a 5 MHz transducer at a sweep speed of 300 mm/s and sample gate of 1 mm. The following echocardiographic variables were measured as right heart functional evaluation: Tricuspid annular velocity, tricuspid annular plane systolic excursion (TAPSE) and right ventricular fractional area of change (FAC). Tricuspid annular velocity and Mitral annular velocity were measured using Tissue Doppler Imaging from apical 4-chamber view. TAPSE was measured from an M-mode recording of the lateral aspect of the tricuspid valve annulus seen from the left parasternal apical 4-chamber view. The sample volume was placed on the septum side and the Right free wall side [25]. FAC was defined using the formula (end-diastolic area − end-systolic area)/end-diastolic area × 100) and assessed from the apical 4-chamber view [26]. Peak velocity of the right ventricular outflow tract (RVOT), ACT (acceleration time) and ET (Ejection time) were measured in the parasternal short-axis view with the sample volume placed at the annulus of the pulmonary valve using pulsed-wave Doppler [27]. In the short-axis view, the left fractional shortening (FS) was calculated as follow:

FS (%) = (Left ventricular end-diastolic diameter (LVIDd) − Left ventricular end-systolic diameter (LVDs))/LVDd × 100.

Continuous-wave Doppler was used to measuring the peak velocity of the tricuspid regurgitant (TR) in the apical 4-chamber view [28]. Well visualized and regular shape and contour of TR waves were selected in our study.

### 2.7. Statistical Analysis

All data were analyzed using statistical software SPSS version 28 (Statistical Package for Social Science; International Business Machines Corporation, Chicago, IL, USA). The data were expressed as mean ± standard deviation. Categorical data are expressed as numbers and percentages. For normally distributed parameters, differences between groups were analyzed using a one-way ANOVA followed by post hoc analysis with Bonferroni correction. For non-parametric parameters, differences between groups were evaluated using a non-parametric Kruskal–Wallis test followed by post hoc analysis with Dunn’s multiple comparison test and box plotted as median and interquartile range. Pearson’s correlation coefficients and multivariate linear regression analysis were used to assess the relationship between PAWR variables and hemodynamic variables and echocardiographic right heart functional variables.

## 3. Results

### 3.1. Effect of APE on Hemodynamics, Echocardiographic and PAWR Variables

**Figure 3:**
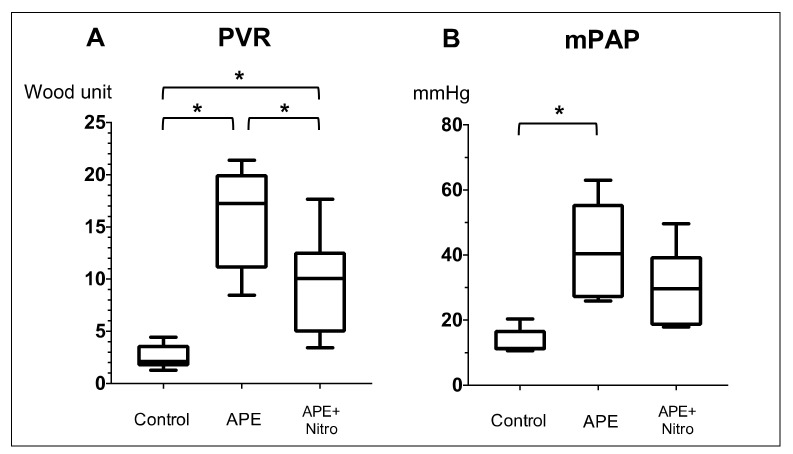
Whisker box plots showing the median (central horizontal line) and interquartile range (25% downward, 75% upward) of the pulmonary vascular resistance (PVR) (**A**) and mean pulmonary artery pressure (mPAP) (**B**) at the baseline (control), after induction of acute pulmonary embolism (APE) and after APE and treatment with nitroprusside (APE + nitro). The upper and lower ends of the whiskers indicate the maximum and minimum values. * *p* < 0.05.

**Figure 4:**
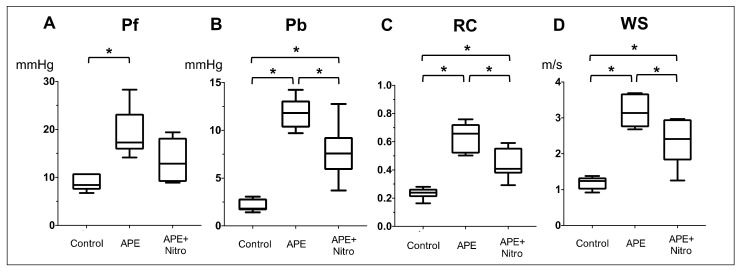
Whisker box plots showing the median and interquartile range of the pulmonary artery wave reflection (PAWR) parameters at the baseline, after induction of acute pulmonary embolism (APE) and after APE and treatment with nitroprusside (APE + nitro). (**A**) forward-traveling pressure (Pf), (**B**) backward-traveling pressure (Pb), (**C**) reflection coefficient (RC), (**D**) wave speed (WS). * *p* < 0.05.

Hemodynamic, Echocardiographic and PAWR variables of all dogs were summarized (Table 1, Figure 3 and Figure 4). Regarding hemodynamic and echocardiographic variables, dextran microsphere injection significantly increases PVR from 2.5 ± 1.2 to 16 ± 5.0 Woods unit (Figure 3A, *p* = 0.0017), mPAP from 13 ± 4 to 42 ± 15 mmHg (Figure 3B, *p* = 0.009) and TR flow from 217.7 ± 32.2 to 381 ± 63 (Table 1, *p* = 0.001). On the other hand, there was a significant reduction in CO from 2.75 ± 0.1 to 2.2 ± 0.4 (*p* = 0.017) and EtCO from 43 ± 4.0 to 35 ± 2 (*p* = 0.02). TAPSE and FAC are also significantly decreased (TAPSE: from 11.4 ± 2.0 to 9.5 ± 1.0, *p* = 0.04; FAC: from 45.4 ± 14.3 to 28.2 ± 11, *p* = 0.02). The injection of dextran microsphere also significantly increases Pf from 8.8 ± 1.7 to 19.2 ± 5.1 (Figure 4A, *p* = 0.02), Pb from 2.1 ± 0.67 to 11.8 ± 1.7 (Figure 4B, *p* = 0.0001), RC from 0.23 ± 0.04 to 0.64 ± 0.1 (Figure 4C, *p* < 0.0001) and WS from 1.2 ± 0.2 to 3.18 ± 0.49 (Figure 4D, *p* = 0.0005). In particular, Pb was increased 5.5 times with the development of PH.

### 3.2. Changes in Hemodynamics, Echocardiographic and PAWR Variables after Nitroprusside Administration

From the result of Table 1, Figure 3 and Figure 4, nitroprusside significantly decreases PVR from 16 ± 5.0 to 9.6 ± 5 Woods unit (*p* = 0.02) and MAP from 91 ± 21 to 75 ± 12 in dogs with APE. However, there was no significant difference in mPAP, CO, EtCO_2_, TAPSE, FAC and TR flow after administration of nitroprusside. On the other hand, nitroprusside significantly decreases Pb from 11.8 ± 1.7 to 6.0 ± 2.3 (*p* = 0.039), RC from 0.64 ± 0.1 to 0.44 ± 0.11 (*p* = 0.0048) and WS from 3.18 ± 0.49 to 2.33 ± 0.65 (*p* = 0.012).

### 3.3. Correlation between PAWR Variables and Hemodynamic and Echocardiographic Right Heart Functional Parameters

**Figure 5:**
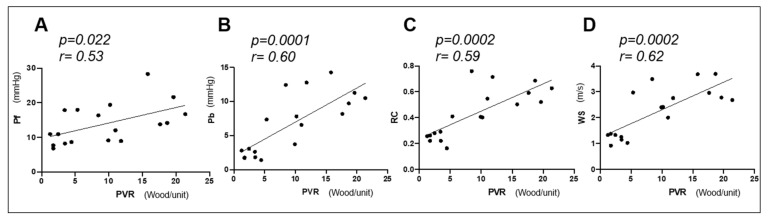
Correlation between pulmonary vascular resistance (PVR) and pulmonary artery wave reflection (PAWR) variables (Pf (**A**), forward-traveling pressure; Pb (**B**), backward-traveling pressure; RC (**C**), reflection coefficient; WS (**D**), wave speed). r, the correlation coefficient. There was a significant positive correlation between PVR and PAWR parameters (*p* < 0.05).

Table 2 summarizes the correlation results between PAWR variables and hemodynamic variables and variables of right heart functional evaluation. There was a statistically significant positive correlation between PVR and Pf, Pb, RC, WS (Table 2 and Figure 5): PVR vs. Pf (r = 0.53; *p* = 0.022), PVR vs. Pb (r = 0.60; *p* = 0.0001), PVR vs. RC (r = 0.59; *p* = 0.0002) and PVR vs. WS (r = 0.62; *p* = 0.0002). Similarly, mPAP showed a significant positive correlation with Pf, Pb, RC and WS (Table 2). CO was negatively correlated with Pf only (r = −0.56, *p* = 0.015).

**Figure 6:**
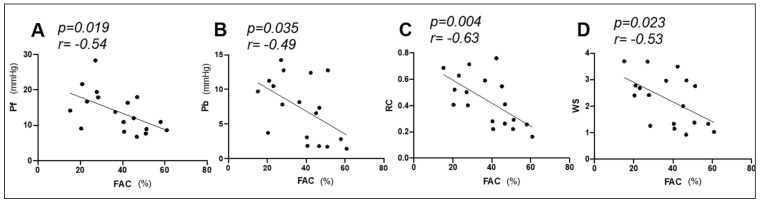
Correlation between pulmonary artery wave reflection (PAWR) parameters (Pf (**A**), forward-traveling pressure; Pb (**B**), backward-traveling pressure; RC (**C**), reflection coefficient; WS (**D**), wave speed) and right ventricular fractional area of change (FAC). r, the correlation coefficient. There was a significant reverse correlation between PAWR parameters (A, Pf; B, Pb; C, RC; D, WS) and FAC (*p* < 0.05).

FAC was the only right heart function variables that correlated with PVR, which is an indicator for assessing right ventricular afterload (r = −0.67; *p* = 0.002). Therefore, we investigated relationship between PAWR variables and FAC. FAC was negatively correlated with PAWR variables (Table 2 and Figure 6): FAC vs. Pf (r = −0.54; *p* = 0.019), FAC vs. Pb (r = 0.49; *p* = 0.032), FAC vs. RC (r = −0.63; *p* = 0.004), FAC vs. WS (r = −0.53; *p* = 0.023).

### 3.4. Effect of PAWR Variables on Hemodynamic and Echocardiographic Right Heart Functional Parameters

Result of linear regression analysis of variables of PAWR, hemodynamic parameters and echocardiographic right heart functional parameters are shown in Table 3. There was a significant effect of Pf on PVR, mPAP, CO and FAC (R^2^ = 0.29, 0.22, 0.31, 0.3; *p* < 0.05), respectively. Pb exhibited significant effect on PVR, mPAP and FAC (R^2^ = 0.42, 0.33, 0.25, *p* < 0.05), respectively. RC demonstrated significant effect on PVR, mPAP and FAC (R^2^ = 0.36, 0.41, 0.4; *p* < 0.05), respectively. WS showed a significant effect on PVR, MPAP and FAC (R^2^ = 0.59, 0.48, 0.28; *p* < 0.05).

## 4. Discussion

This study investigated the characteristics of PAWR through experimental induction of APE in dogs and then measuring the PAWR before and after administration of vasodilator. We also examined the relationship between PAWR and hemodynamic variables and right ventricular function.

It has been reported that PAWR is significantly increased in PH patients [13,29,30]. PAWR is generated when the vascular impedance changes between the proximal and distal vasculature due to vascular remodeling, arteriosclerosis and thrombus [13,31,32,33]. Su et al. have discussed the changes in PAWR in patients with PH [13]. As PH develops, the pulmonary artery becomes a high-pressure, high-resistance, low compliance and capacitance [9,34,35]. In patients with pulmonary hypertension, Pf increased compensatory due to increased afterload in the pulmonary artery and Pb and wave speed are also increased due to differences in vascular impedance between the proximal and distal vasculature, showing a clear difference from the healthy group [36]. In the present study, dogs developed PH after microsphere injection, which revealed the establishment of our model and PAWR was increased significantly along with PAP and PVR. The PAWR was amplified due to the impedance mismatch between the proximal pulmonary blood vessel and the distal pulmonary blood vessel caused by the clogging of microspheres to the peripheral pulmonary arteries [37,38,39]. FAC and TAPSE were significantly reduced after microsphere injection, indicating that excessive increase in right ventricular afterload led to decreased right ventricular function.

In the current study, we also investigated the changes of hemodynamics, echocardiographic and PAWR variables after administration of nitroprusside as a vasodilator. Nitroprusside is not a specific therapeutic medication that targeting pulmonary artery vascular. By deliberately using nitroprusside and mildly lowering the pulmonary artery, we investigated which parameter most accurately represents the condition. After administration of nitroprusside, PAP tended to decrease but not significantly. On the other hand, PVR and PAWR variables were significantly decreased after nitroprusside administration. These results indicated that PVR and PAWR may be able to capture subtle changes of right ventricular afterload that cannot be captured by PAP. Other drugs, for example, Terlipressin, which in a porcine model seemed to have better properties in an acute situation, can be used to save time for diagnosis and treatment [40].

PVR is commonly used as an indicator of right ventricular afterload [6,41]. Recently, some studies reported that PAWR is closely related to the right ventricular afterload [13,36,42]. The present study also indicated that the PAWR may be a useful indicator for assessing right ventricular afterload because PVR and PAWR were significantly correlated. More interestingly, the significant correlation and regression between PAWR and FAC suggested that PAWR may be related to right ventricular function.

Su et al. have discussed the PAWR after persisted pulmonary endarterectomy (PEA) in patients with chronic thromboembolic pulmonary hypertension (CTEPH) [36]. In their report, although PVR decreased substantially following PEA, there was some patient who has large PAWR persisted. Persistent wave reflection indicates a lack of improvement in vascular impedance mismatch and may contribute to the persistence of symptoms in some patients. Therefore, PAWR may provide novel insights into pulmonary arterial hemodynamics, which is different from PVR. PAWR is not only correlated with PVR but also the right ventricular function parameter, which denotes that PAWR may be useful as a new evaluation method in PH, considering ventricular-arterial coupling (V-A coupling).

PH remains a high mortality disease [43]. One of the reasons may be the delay in diagnosis [35]. The normal pulmonary circulation is a low-pressure, high-compliance system with a large vascular reserve in the form of nonperfused vessels [44]. Consequently, increased mPAP occurs relatively late in the progression of disease when damage to the vasculature is advanced [45]. Therefore, mPAP may not reveal the true severity of the pulmonary vascular disease. Previous studies have reported that PAWR is useful as an early diagnostic marker for PH [13]. In our study as well, PAWR may be useful as an early diagnostic index for PH, taking into account the possibility of capturing subtle changes in right ventricular afterload that cannot be captured by PAP in PH model dogs after administration of vasodilator. The measurement of PAWR may be limited in clinical settings at present because of invasive. However, with the continuous innovation of medical imaging tools, recent advances in imaging tools hopefully facilitate future use of wave intensity analysis in pulmonary hypertension [31].

### Limitations

Some of the statistical comparisons may be underpowered because the number of dog samples used in this study was small. All dogs used in this study were female. However, female dogs with comparable numbers have been previously reported [46]. Unlikely, the distribution of men and women may affect the characteristics of PAWR. The anesthesia can also affect the outcome. However, in this study, anesthesia of all dogs was performed under the same conditions and all derived measurements have been collected when stable hemodynamics was achieved. Nitroprusside is not a drug that works selectively on pulmonary artery blood vessels. Hence, we need to verify whether a drug that selectively dilates the pulmonary artery will produce similar results. There are various causes of PH and the PH model in this validation study has hemodynamics similar to APE. However, the use of autologous blood thrombi could have more natural than dextran microspheres [47]. Therefore, it is unclear whether this result can be applied to PH caused by other cardiovascular or respiratory morbidities.

## 5. Conclusions

The present study is the first paper to measure PAWR in a dog model of APE using dual-tipped sensor wire. As a result of investigating the characteristics of PAWR, PAWR may detect changes related to right ventricular afterload earlier than PAP. PAWR provides novel information about pulmonary vascular disease and is expected to contribute to PH patients for the clinical setting.

## Figures and Tables

**Table 1:** Hemodynamic, echocardiographic and pulmonary artery wave reflection variables.

Hemodynamic Variables	Control	APE	APE + Nitro
HR, /min	128 ± 9	138 ± 11	132 ± 13
BT, °C	35.5 ± 1.0	36 ± 1	36 ± 1
sPO_2_, %	99 ± 1	98 ± 1	99 ± 1.4
EtCO_2_, mmHg	43 ± 4.0	35 ± 2 *	36 ± 2 **
SAP, mmHg	109 ± 9	105 ± 17	91 ± 12 **†
MAP, mmHg	95 ± 13.0	91 ± 21	75 ± 12 **†
DAP, mmHg	86 ± 17	82 ± 22	68 ± 12 **†
RAP, mmHg	4 ± 1	6 ± 3	6 ± 3
LAP, mmHg	7 ± 1	7 ± 2	7 ± 2
SPAP, mmHg	20 ± 4	59 ± 13 *	41 ± 13 **†
mPAP, mmHg	13 ± 4	42 ± 15 *	30 ± 12
DPAP, mmHg	10 ± 4	33 ± 16 *	25 ± 12
CO, L/min	2.75 ± 0.1	2.2 ± 0.4 *	2.4 ± 0.6
SV, mL	21.5 ± 1.4	16 ± 2.5 *	18.5 ± 10.1
PVR, wood unit	2.5 ± 1.2	16 ± 5.0 *	9.6 ± 5 **†
**Echocardiographic Variables**			
RVOT flow peak velocity, cm/s	82.0 ± 12.7	74 ± 15	77 ± 15
Pulmonary ET, ms	203 ± 26	187 ± 27	178 ± 10
Pulmonary ACT, ms	96.7 ± 26.5	67 ± 14	82 ± 11
Pulmonary ACT/ET	0.47 ± 0.10	0.37 ± 0.1	0.46 ± 0.08
LVFS, %	33.6 ± 7.5	43.9 ± 12.5	39 ± 8.5
LVIDd, mm	26.4 ± 3.3	22.8 ± 2.36	24.9 ± 3.2
TAPSE, mm	11.4 ± 2.0	9.5 ± 1.0 *	9.6 ± 2.2
Tricuspid annular velocity Fw S’, cm/s	7.9 ± 2.6	8.7 ± 2.2	7.7 ± 3.0
Tricuspid annular velocity Fw E’, cm/s	7.7 ± 1.8	6.9 ± 1.8	6.9 ± 3.0
Tricuspid annular velocity Fw A’, cm/s	7.7 ± 1.7	9.6 ± 3.2	9.6 ± 3.4
LVOT peak velocity, cm/s	81.5 ± 18.0	86.3 ± 22.2	86 ± 21
FAC, %	45.4 ± 14.3	28.2 ± 11 *	38 ± 12
TR, cm/s	217.7 ± 32.2	381 ± 63 *	295 ± 40 **
PAWR variables			
Pf, mmHg	8.8 ± 1.7	19.2 ± 5.1 *	13.5 ± 4.4
Pb, mmHg	2.1 ± 0.67	11.8 ± 1.7 *	6.0 ± 2.3 **†
RC (Pb/Pf)	0.23 ± 0.04	0.64 ± 0.1 *	0.44 ± 0.11 **†
WS, m/s	1.2 ± 0.2	3.18 ± 0.49 *	2.33 ± 0.65 **†

Hemodynamic, echocardiographic and pulmonary artery wave reflection parameters in a dog model of acute pulmonary embolism. Data expressed as Mean ± SD. The data represent the comparison between the derived parameters at the baseline after induction of anesthesia (control), after experimental induction of APE and later on after treatment with Nitroprusside (APE + Nitro). * control vs. APE (*p* < 0.05); ** APE vs. APE + Nitro; †, control vs. APE + Nitro. Abbreviations: HR, heart rate; BT, body temperature; sPO_2_, oxygen saturation of peripheral artery; EtCO_2_, end-tidal CO_2_ concentration; SAP, systemic arterial pressure; MAP, mean arterial pressure; DAP, diastolic arterial pressure; RAP, right atrial pressure; LAP, left atrial pressure; SPAP, systolic pulmonary arterial pressure; mPAP, mean pulmonary arterial pressure; DPAP, diastolic pulmonary arterial pressure; CO, cardiac output; SV, stroke volume; PVR, pulmonary vascular resistance; RVOT, right ventricular outflow tract; ET, ejection time; ACT, acceleration time; LVFS, left ventricular fractional shortening; LVIDd, Left ventricular internal diameter end diastole; TAPSE, tricuspid annular plane systolic excursion; Fw, free wall; LVOT, left ventricular outflow tract; FAC, right ventricular fractional area of change; TR, tricuspid regurgitation; Pf, forward-travelling pressure; Pb, backward-travelling pressure; RC, reflection coefficient; WS, wave speed.

**Table 2:** Correlation between PAWR indices and hemodynamic and echocardiographic variables.

Variables	Pf		Pb		RC		WS	
	R	*p*-Value	R	*p*-Value	R	*p*-Value	R	*p*-Value
PVR	0.53 *	0.022	0.6 *	0.0001	0.59 *	0.0002	0.62 *	0.0002
mPAP	0.47 *	0.048	0.57 *	0.013	0.65 *	0.004	0.69 *	0.002
CO	−0.56 *	0.015 *	−0.31	0.2	−0.41	0.089	−0.3	0.23
RAP	0.015	0.95	0.078	0.79	0.27	0.28	0.186	0.46
LAP	−0.053	0.83	−0.15	0.54	−0.221	0.38	−0.049	0.85
MAP	0.063	0.81	−0.06	0.8	−0.012	0.96	0.034	0.89
FAC	−0.54 *	0.019	−0.49 *	0.035	−0.63 *	0.004	−0.53 *	0.023
TAPSE	−0.35	0.15	−0.21	0.4	−0.63	0.17	−0.39	0.11

Pearson’s correlation between PAWR variables (Pf, forward-traveling pressure; Pb, backward-traveling pressure; RC, reflection coefficient; WS, wave speed) and hemodynamic variables and echocardiographic right heart functional variables. Abbreviations: MAP, mean arterial pressure; RAP, right atrial pressure; LAP, left atrial pressure; mPAP, mean pulmonary arterial pressure; CO, cardiac output; PVR, pulmonary vascular resistance; FAC, right ventricular fractional area of change; TAPSE, tricuspid annular plane systolic excursion. * *p* < 0.05.

**Table 3:** Effect of PAWR indices on hemodynamic and echocardiographic variables.

Variables	Pf		Pb		RC		WS	
	R^2^	*p*-Value	R^2^	*p*-Value	R^2^	*p*-Value	R^2^	*p*-Value
PVR	0.29 *	0.022	0.42 *	0.004	0.36 *	0.008	0.59 *	<0.001
mPAP	0.22 *	0.048	0.33 *	0.013	0.41 *	0.004	0.48 *	0.002
CO	0.31 *	0.015	0.1	0.2	0.17	0.089	0.089	0.23
RAP	0.00023	0.95	0.006	0.75	0.072	0.28	0.034	0.46
LAP	0.0028	0.83	0.024	0.53	0.049	0.38	0.0023	0.85
MAP	0.004	0.8	0.004	0.8	0.00015	0.96	0.0011	0.89
FAC	0.3 *	0.02	0.25 *	0.035	0.4 *	0.005	0.28 *	0.02
TAPSE	0.12	0.15	0.044	0.4	0.15	0.1163	0.15	0.1105

Linear regression between PAWR variables (Pf, forward-traveling pressure; Pb, backward-traveling pressure; RC, reflection coefficient; WS, wave speed) and hemodynamic variables and echocardiographic right heart functional variables. Abbreviations: MAP, mean arterial pressure; RAP, right atrial pressure; LAP, left atrial pressure; MPAP, mean pulmonary arterial pressure; CO, cardiac output; PVR, pulmonary vascular resistance; FAC, right ventricular fractional area of change; TAPSE, tricuspid annular plane systolic excursion. * *p* < 0.05.

## Data Availability

The data presented in this study are available on request.
